# Comparative Effects of Dry and Wet Aging on Quality, Flavor Development, and Metabolomic Profiles in Grouper Fillets

**DOI:** 10.1155/anu/3310290

**Published:** 2026-04-23

**Authors:** Qin-Shan Yu, Hsiu-Ming Liu, Huey-Jine Chai, Hsin-Hui Su, Yung-Tsung Chen

**Affiliations:** ^1^ Department of Food Science, National Taiwan Ocean University, Keelung, 202301, Taiwan, ntou.edu.tw; ^2^ Fisheries Research Institute, Ministry of Agriculture, Keelung, 202301, Taiwan, tfrin.gov.tw

**Keywords:** aging techniques, flavor development, grouper fillets, metabolomics

## Abstract

Aging techniques are widely used to enhance meat quality; however, they are seldom applied to fish. This study investigated the effects of low‐temperature dry and wet aging on the quality, flavor development, and metabolomic profiles of grouper (*Epinephelus lanceolatus* × *E. fuscoguttatus*) fillets. The fish fillets were aged at 2°C ± 1°C for 8 days, with samples collected every 2 days. Dry‐aged fillets showed progressive dehydration and weight loss but improved water‐holding capacity (WHC) and reduced cooking loss. By contrast, wet‐aged fillets retained moisture but had higher cooking loss. Both methods showed initial pH increases followed by declines. Thiobarbituric acid reactive substances (TBARS) and volatile basic nitrogen (VBN) increased during aging but remained within acceptable limits. Dry‐aged fillets had the highest free amino acid (FAA) content on day 4, with glutamic acid and arginine as major contributors. Inosinic acid significantly increased on day 2, supporting flavor improvement. Metabolomic analysis identified mannitol and pyridoxine as characteristic metabolites in dry aging and L‐aspartate and pyroglutamic acid in wet aging. Our results indicated that both aging processes induce distinct metabolic pathways that contribute to flavor enhancement, alterations in the nutritional profile, and potential risks related to oxidative deterioration and food safety. In conclusion, aging improved grouper fillet quality through distinct biochemical mechanisms that promote the formation of flavor compounds. These findings demonstrate that aging is a promising approach for accelerating flavor development and value to fish products.

## 1. Introduction

Fish meat is an important source of animal protein and is widely consumed for its health benefits. Among various species, the grouper is highly valued in aquaculture for its rapid growth, high‐quality meat, and high economic value. Its production is mainly located in Asia, making it one of the most extensively cultured and economically important fish species in this region. In addition to high‐quality protein, grouper is also rich in omega‐3 fatty acids. However, as living standards improve, consumers not only focus on nutritional value but also demand better flavor in meat products. Unlike red meat, the high endogenous enzymatic activity in fish muscle [1] can accelerate flavor development in a short period. Furthermore, as consumer preference for natural and additive‐free food increases, aging technology has become an effective and practical strategy for processing high‐quality fish products.

Dry‐ and wet‐aging techniques are widely employed in meat processing. Aging is a method that leverages the activity of endogenous enzymes to degrade muscle proteins, thereby improving tenderness and enhancing sensory properties [2]. This enhanced tenderness is mainly attributed to hydrolytic activity, which leads to the accumulation of taste‐active peptides, free amino acids (FAAs), and nucleotides [2]. While aging methods have been extensively studied in red meats, such as beef and pork, recent research shows that dry‐aged processing yields higher levels of volatile flavor compounds in beef loin [3]. The application of aging to aquatic meat products remains underexplored.

Compared to red meat, fish muscle has higher water activity and a higher content of polyunsaturated fatty acids (PUFAs), making it more susceptible to oxidation. Furthermore, lipid oxidation and microbial spoilage rates are rapid in fish, making it highly sensitive to aging conditions [4]. Currently, studies on postmortem fish primarily focus on preserving freshness [4, 5], whereas the application of dry aging to enhance flavor remains limited. Previous research on postmortem storage has mostly focused on fatty fish species, such as trout [5] and tuna [6], emphasizing texture softening rather than flavor development. In addition, a previous study investigated the use of dry aging to delay spoilage in monkfish during cold storage [7]. However, few comprehensive studies have compared the metabolomic mechanisms of dry and wet aging in white fish. Specifically, it remains unclear how different aging environments affect the profiles of flavor‐related metabolites, aside from protein degradation. Therefore, this study aimed not only to analyze fish texture but also the levels of flavor compounds while evaluating the balance between flavor enhancement and spoilage risk.

Therefore, this study investigated the effects of low‐temperature dry and wet aging on quality, flavor development, and metabolite changes in grouper fillets. We aimed to provide insights into flavor development in aged fish and to evaluate correlations among meat quality, flavor development, and aging‐derived metabolites.

## 2. Materials and Methods

### 2.1. Preparation and Aging of Grouper Fillets

A total of 14 fresh groupers (*Epinephelus lanceolatus* × *Epinephelus fuscoguttatus*), each weighing approximately 1 kg, were purchased from a local seafood market in Keelung, Taiwan, and euthanized using the ikejime method. Fillets were obtained from both sides of each fish following standard processing procedures, resulting in 27 fillets from the dorsal muscle, each weighing approximately 150 g and about 2 cm thick. To reduce variation due to individual differences, the fillets were randomly assigned to treatment groups. Three fillets were used for the unaged group (day 0). The remaining 24 fillets were evenly split into dry‐aging and wet‐aging groups, with three fillets (*n* = 3) randomly allocated for sampling on each designated day (days 2, 4, 6, and 8). Fillets in the wet‐aged group were vacuum‐sealed. Both dry‐aged and wet‐aged fillet samples were aged in an aging chiller (LT‐100DA, BO RUEI Technical Co., Ltd., Taichung, Taiwan) at 2.0°C ± 1.0 °C and 75% ± 10% relative humidity for 8 days. The cabinet was sanitized beforehand. On days 2, 4, 6, and 8, three fillet samples from each group were collected for physicochemical and microbiological analyses. The fillets were rotated every 2 days to ensure even exposure to the aging environment. Afterward, the fillets were stored at −80 °C for subsequent analysis.

### 2.2. Proximate Chemical Composition Analyses

Proximate chemical composition (moisture, protein, lipid, and ash content) was determined following AOAC procedures [8], with adaptations. Duplicate measurements were obtained, and the mean values were used for subsequent statistical analyses.

### 2.3. pH Determination

Approximately 5 g of fish meat was collected following sampling and homogenized with 45 mL of distilled water (1:10 w/v) using a stomacher (BagMixer 400, Interscience, Saint‐Nom‐la‐Bretèche, France). The pH of the homogenate was immediately measured with a pH meter (pH 510, Eutech Instruments, Singapore).

### 2.4. Total Plate Count (TPC)

The meat homogenate was serially diluted with sterile saline, and the TPC was determined using plate count agar (Cat. NCM0010A, Neogen Corporation, Lansing, USA). The plates were incubated at 37 °C for 48 h to determine total viable counts, which were expressed as log CFU/g of meat weight.

### 2.5. CIELAB Color Space

The color parameters (CIE *L* ^∗^, *a* ^∗^, and *b* ^∗^) of each fish fillet (~2 × 2 × 2 cm) were measured using a color spectrophotometer (TC‐1800 MK‐II, Nippon Denshoku Industries Co., Ltd., Tokyo, Japan). The color difference (Δ*E*) was calculated employing the formula described by Liu et al. [9].

### 2.6. Texture Profile Analysis (TPA)

Texture was evaluated using a modified protocol based on Kocatepe et al. [10]. Briefly, TPA was conducted on fish fillet cubes (2 × 2 × 2 cm), using a texture analyzer (RapidTA+, Horn Instruments Co. Ltd., Taoyuan, Taiwan). The TPA parameters were hardness (N), adhesiveness (mJ), resilience, springiness (cm), cohesiveness, gumminess (N), and chewiness (N·cm). Each sample was tested in triplicate, and mean values were used for statistical analysis.

### 2.7. Water‐Holding Capacity (WHC)

The WHC of fish meat was determined using the released water method described by Joo [11], with modifications. Approximately 5 g of intact fish meat was placed on a preweighed filter paper (ADVANTEC No. 1, 90 mm diameter) and covered with another piece of the same filter paper. A pressure of 1.0 kg was applied to the sample and held for 5 min at room temperature. The wet filter paper was immediately weighed after removing the compressed fish fillet.

### 2.8. Cooking Loss

The cooking loss of fish meat was determined using the method of Sun et al. [12], with modifications. Approximately 5 g of fish fillet was weighed and placed into a Ziplock bag and then heated in a water bath at 85 °C for 15 min. Next, the sample was weighed again. Each measurement was performed in triplicate.

### 2.9. Thiobarbituric Acid Reactive Substance (TBARS) Assay

Lipid oxidation was evaluated using the method of Przybylski et al. [13], with modifications. Briefly, 2 g of fish fillet was combined with 5 mL of 10% trichloroacetic acid (TCA) (Cat. T6399, Sigma–Aldrich Corp, St. Louis, MO, USA) in a centrifuge tube and thoroughly homogenized. Then, 5 mL of 0.02 M thiobarbituric acid (Cat. T0192, Tokyo Kasei Kogyo Co., Ltd., Kawaguchi, Japan) solution was added, and the mixture was homogenized again. The sample was centrifuged at 3000 rpm for 10 min, and the resulting supernatant was filtered into a clean centrifuge tube, covered with aluminum foil, and heated in a boiling water bath for 35 min to facilitate color development. After cooling to room temperature, absorbance was measured at 532 nm, using an ELISA reader (Synergy H1, BioTek Instruments, Winooski, VT, USA). The results were expressed as malondialdehyde (MDA) content in mg/kg of meat. Each fish fillet sample was analyzed in triplicate.

### 2.10. Volatile Basic Nitrogen (VBN)

The VBN content was determined following the method of Neri et al. [14], with modifications. Fish meat (2 g) was homogenized with 20 mL of 2.2% TCA, and the filtrate was collected as the test extract. VBN was quantified using a Conway dish, where boric acid served as the absorption solution and saturated potassium carbonate as the alkaline reagent. The sealed dishes were incubated at 37 °C for 90 min to allow volatile bases to diffuse, followed by titration with standardized 0.02 N HCl. A blank prepared with deionized water was assessed under the same conditions.

### 2.11. K‐Value

The K‐value was determined employing the method of Sallam [15], with modifications. ATP‐related compounds (ATP, ADP, AMP, IMP, inosine, and hypoxanthine) were extracted from 2 g of fish fillet with 6% perchloric acid, neutralized to pH 6.5 with KOH, and adjusted to a final volume of 20 mL. After filtration through a 0.22 µm membrane, 10 µL of the extract was analyzed using a high‐performance liquid chromatography (HPLC) system (LC‐2040C Plus, Shimadzu, Kyoto, Japan) equipped with a CAPCELL PAK C18 AQ column (150 × 4.6 mm, 5 µm). The mobile phase consisted of 50 mM KH_2_PO_4_ (pH 6.5) and methanol at a flow rate of 0.6 mL/min and UV detection at 254 nm.

### 2.12. FAA Analysis

FAAs were extracted from 2 g of fish fillet, using 5% TCA per the method of Zhang et al. [16], with modifications. The supernatants obtained after homogenization and centrifugation were diluted and filtered through a 0.22 μm polyvinylidene fluoride membrane. FAAs were determined by HPLC (LC‐2040C 3D Plus, Shimadzu) after precolumn derivatization with phenylisothiocyanate. A Diamonsil AAA column (4.6 × 250 mm, Dikma Technologies, CA, USA), kept at 45 °C, was used to separate the compounds. The mobile phase was a binary system composed of sodium acetate buffer and acetonitrile/methanol/water at a flow rate of 0.6 mL/min. The amino acids were identified at 254 nm. The taste activity value (TAV) was calculated as the ratio of the concentration of each amino acid to its respective taste threshold [17], where TAV > 1 indicates a significant contribution to taste.

### 2.13. TCA‐Soluble Peptides

TCA‐soluble peptides were measured using the Folin‐phenol method, as described by Lan et al. [18]. Briefly, 5 g of minced fish fillet was mixed with 10 mL of 10% TCA and centrifuged at 13,500 rpm for 10 min. The resulting supernatant was combined with the Folin‐phenol reagent, and the response was recorded. The concentration of TCA‐soluble peptides was expressed as μmol tyrosine per gram of meat, based on a standard curve generated using bovine serum albumin.

### 2.14. Sodium Dodecyl Sulfate Polyacrylamide Gel Electrophoresis (SDS‐PAGE)

SDS‐PAGE was performed following the method of Bao et al. [19], with minor modifications. Briefly, 1.25 g of fish fillet was homogenized with 25 mL of ice‐cold distilled water at 13,500 rpm for 2 min. The resulting homogenate was mixed with an equal volume of 4× SDS sample buffer containing 5% β‐mercaptoethanol. The mixture was heated at 100 °C for 5 min and centrifuged to obtain the supernatant. The protein concentration was determined using a BCA Protein Assay Kit (T‐Pro Biotechnology, New Taipei City, Taiwan), following the manufacturer’s instructions. Equal amounts of protein (5 μg/µL) were loaded into each well and separated via SDS‐PAGE, using a 5% stacking gel and a 12% resolving gel. Following electrophoresis, the gels were stained with One‐Step Blue protein gel stain (Biotium, Inc., Fremont, CA, USA) for 1 h and destained in distilled water for ~20 h.

### 2.15. Metabolomics Analysis

Untargeted metabolomic analysis was conducted by the National Human Microbiome Core Facility, as described by Kao et al. [20]. Polar and nonpolar metabolites were extracted from 100 mg of homogenized freeze‐dried fish fillets with 80% methanol, followed by centrifugation (15,000× g, 5 min, and 4 °C). The supernatant was filtered through a 0.22 µm membrane and stored at −20°C until analysis.

Metabolomic profiling was performed on an Agilent 1290 UHPLC system coupled with a Bruker maXis UHR‐TOF mass spectrometer (Bruker Daltonics, Bremen, Germany). Polar metabolites were separated on a BEH (ethylene bridged hybrid) amide column (2.1 × 100 mm, 1.7 µm; Waters Corporation, Milford, MA, USA) using ammonium acetate/formic acid in water and acetonitrile as the mobile phases. The column was maintained at 40°C, the injection volume was 10 µL, and electrospray ionization was operated in the positive mode (scan range *m/z* 50–1500). Each sample was analyzed in triplicate, with pooled quality‐control samples and blanks injected throughout the sequence to ensure system stability and data quality.

Raw data obtained from the maXis UHR‐TOF MS were processed using the MS‐DIAL software (version 4.60). Peak detection and alignment were performed with a minimum peak height threshold of 10,000 and a retention time tolerance of 0.2 min. All other parameters were set to default values. Metabolites were identified by matching the MS/MS spectra against the MS‐DIAL metabolomics MSP spectral database.

### 2.16. Fatty Acid Analysis

The analysis of the fatty acid composition was commissioned to Chi Mei Inspection Tech Co., Ltd. (Taiwan). The measurement was performed following the official method MOHWO0014.00, published by the Ministry of Health and Welfare, Taiwan. In brief, the lipids were extracted from the samples and converted into fatty acid methyl esters (FAMEs) through a process of acid hydrolysis and methylation. Approximately 100–200 mg of homogenized fish muscle was mixed with 100 mg of pyrogallic acid, and 1 mL of triheneicosanoin (C21:0) was added to the mixture as an internal standard. The sample was then hydrolyzed using 8.3 M hydrochloric acid in a water bath at 70°C–80°C for 40 min. After cooling, the hydrolyzed lipid fraction was extracted using a solvent mixture of diethyl ether and petroleum ether (1:1, v/v). The organic solvent was subsequently evaporated under reduced pressure at 40°C. The obtained lipid extract was derivatized into FAMEs using a 14% boron trifluoride‐methanol solution at 110°C. The prepared FAMEs were analyzed using a gas chromatograph equipped with a flame ionization detector (GC‐FID). The separation was carried out on a CP‐Sil 88 capillary column (100 m × 0.25 mm i.d. and 0.20 µm film thickness). The GC oven temperature program was set as follows: The initial temperature was held at 170°C for 40 min, then increased to 200°C at a heating rate of 3°C/min, and finally maintained at 200°C for 50 min. The individual fatty acids were identified by comparing their retention times with those of commercial FAME standards. The absolute content of each fatty acid was quantified based on the peak area of the internal standard.

### 2.17. Statistical Analysis

All statistical analyses and graphical illustrations were performed using GraphPad Prism version 9.0 (GraphPad Software, San Diego, CA, USA). Group differences were assessed through one‐way and two‐way analysis of variance (ANOVA), followed by Tukey’s post hoc test for multiple comparisons. Significance was set at *p* < 0.05. Metabolomic data were processed with MetaboAnalyst 6.0, incorporating multivariate approaches, such as principal component analysis (PCA), variable importance in projection (VIP) scoring, volcano plot generation, and correlation heatmap analysis.

## 3. Results

### 3.1. Moisture Loss, Weight Reduction, and Compositional Changes in Grouper Fillets During Dry and Wet Aging

After 8 days of dry aging, the surface of the grouper fillets gradually changed from initially moist to a shrunken, visibly dehydrated state. In contrast, vacuum packaging of the wet‐aged fish fillets prevented moisture loss, allowing the fillets to retain a moist surface even after aging (Figure 1a). Quantitative analysis of weight loss further demonstrated a significant difference between the two aging methods. After 4 days of dry aging, the weight loss of the fish fillets significantly (*p* < 0.0001) increased to 31.14%, accompanied by surface drying but no crust formation. By day 8, weight loss further increased to 44.34%, with noticeable surface shrinkage and partial hardening at the tail. Conversely, wet‐aged fillets showed a 1.16% weight loss on day 8, indicating better moisture retention compared to dry‐aged fillets (Figure 1b).

Figure 1Changes in surface appearance and weight loss of grouper fillets during dry aging (ND) and wet aging (NW). (a) Changes in the surface appearance of grouper fillets during different aging periods and aging methods. (b) Weight loss rate during dry aging and wet aging. Statistical analysis within each aging method was performed using one‐way ANOVA, followed by Dunnett’s multiple comparison test, comparing each time point to day 0.  ^∗∗∗∗^ *p* < 0.0001. For comparisons between different aging methods at the same aging time, independent Student’s *t*‐test were conducted. ^# # # #^ *p* < 0.0001.

**Figure:**
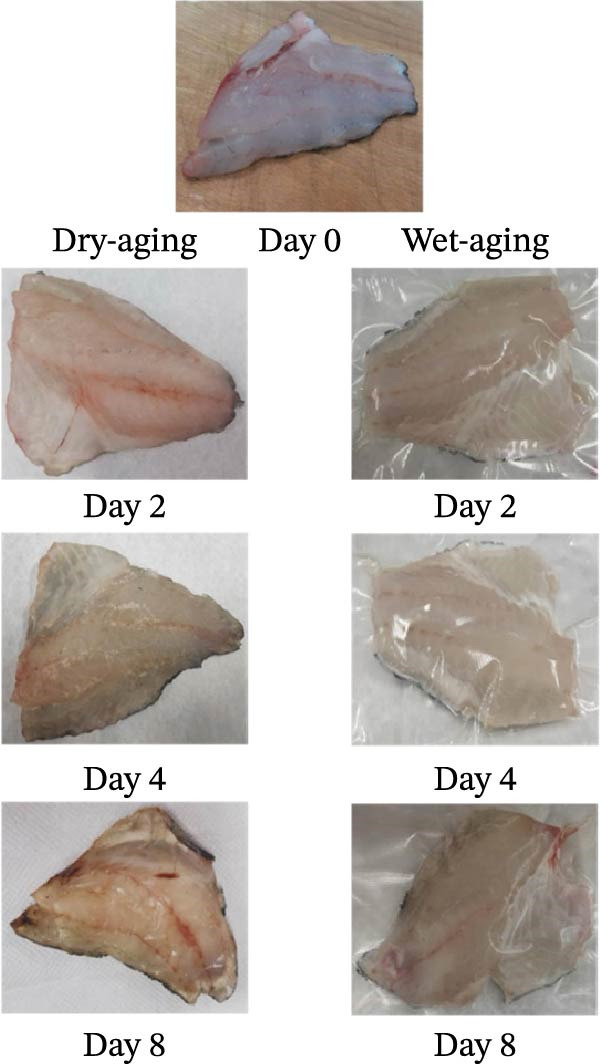
(a)

**Figure:**
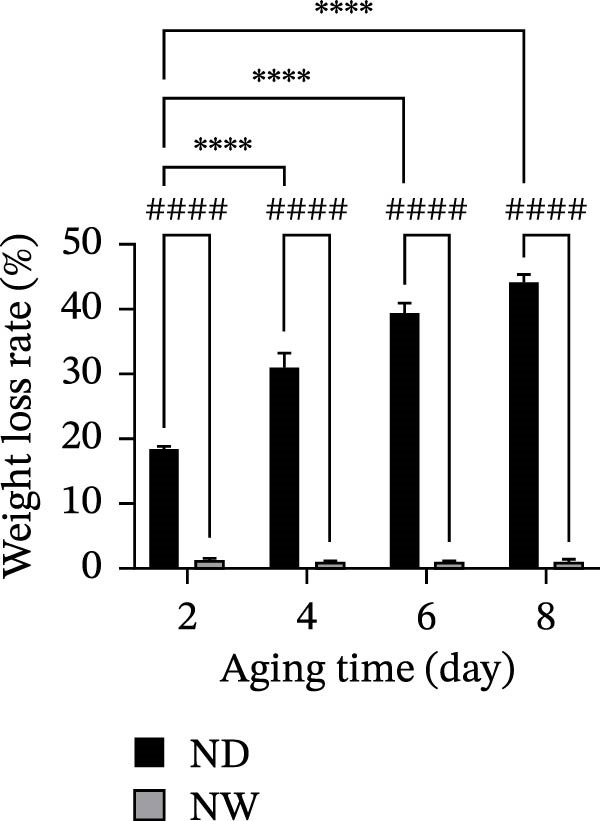
(b)

Additionally, aging treatments changed the chemical composition of the fillets. As dry aging continued, moisture content gradually decreased, dropping to 68.99% by day 6. In contrast, the contents of crude ash, crude protein, and crude fat increased. For wet‐aged fillets, the moisture content dropped to 76.12% by day 6. The crude protein level showed an upward trend, while the crude fat content initially increased and then decreased. Meanwhile, the ash content remained stable (Table 1).

**Table 1:** Changes in proximate composition of grouper fillets during dry aging (ND) and wet aging (NW).

Proximate composition	Aging method	Aging period (day)					Interaction
		0	2	4	6	8	
Moisture (%)	ND	76.65 ± 0.47^a^	72.61 ± 2.36^abB^	70.13 ± 1.65^abB^	68.99 ± 6.72^abA^	70.92 ± 1.17^aB^	*F* (4, 20) = 2.394, *p* = 0.0847
	NW	76.65 ± 0.61^a^	78.62 ± 0.99^abA^	78.00 ± 1.94^abA^	76.12 ± 1.28^abA^	77.29 ± 2.02^aA^	

Crude ash (%)	ND	0.86 ± 0.45^b^	1.45 ± 0.02^abA^	1.31 ± 0.08^abA^	1.67 ± 0.25^a^	1.76 ± 0.20^aA^	*F* (4, 20) = 1.351, *p* = 0.2859
	NW	0.86 ± 0.45	1.17 ± 0.07^B^	1.15 ± 0.03^B^	1.26 ± 0.12	1.19 ± 0.07^B^	

Crude protein (%)	ND	12.51 ± 0.23	12.43 ± 1.27	12.72 ± 1.37	13.10 ± 2.33	14.16 ± 1.60	*F* (4, 20) = 0.0009, *p* = 0.9998
	NW	12.51 ± 0.23	12.69 ± 3.58	12.75 ± 1.94	13.39 ± 1.20	14.20 ± 1.01	

Crude fat (%)	ND	8.76 ± 3.00	12.13 ± 3.10	20.92 ± 10.42	13.23 ± 1.92	10.31 ± 3.14	*F* (4, 20) = 0.6110, *p* = 0.6595
	NW	8.76 ± 3.00	18.54 ± 19.04	13.65 ± 4.61	14.88 ± 7.71	9.00 ± 3.25	

*Note:* Data are expressed as means ± SD (*n* = 3). ^a,b^Different letters within the same row indicate significant differences, as determined by one‐way ANOVA with Tukey’s multiple comparisons test (*p* < 0.05). ^A,B^Different letters in the same column indicate significant differences (*p* < 0.05) between aging methods on the same day, as determined by Student’s *t*‐test. The interaction effects were evaluated using a two‐way ANOVA. The absence of superscript letters indicates no significant differences (*p* > 0.05) as determined by one‐way ANOVA or Student’s *t*‐test.

### 3.2. Changes in Quality, Freshness Indicators, and Color of Grouper Fillets During Dry and Wet Aging

The impact of aging on the quality and freshness of grouper fillets was evaluated by measuring pH, lipid oxidation (TBARS), VBN, TPC, and K‐value. The results demonstrated that, in both methods, the pH of the fish fillets initially increased and subsequently decreased throughout the aging period. The pH of the dry‐aged fish fillets reached 6.51 on day 4. By contrast, the pH of the wet‐aged fish fillets reached 6.58 on day 2, followed by a gradual decline. Both TBARS and VBN values increased progressively with age. After 8 days of dry aging, the TBARS value peaked at 1.63 mg MDA/kg, whereas VBN reached 15.62 mg/100 g on day 6. Similarly, in the wet‐aged samples, the highest TBARS and VBN values indicated 1.08 mg MDA/kg and 11.26 mg/100 g, respectively, on day 8. These values remain within typical acceptable spoilage limits for fish products (30–35 mg/100 g for VBN and 10–20 mg MDA/kg for TBARS) [21, 22], indicating adequate oxidative and microbial safety during the 8‐day aging period.

The TPC increased during the early stages of aging, peaking on day 6 at 3.71 log CFU/g in dry‐aged fillets and 3.73 log CFU/g in wet‐aged fillets, then declined slightly. The K‐value, an indicator of nucleotide degradation, rose continuously, reaching 13.73% in dry‐aged fillets by day 8% and 23.92% in wet‐aged fillets by day 6 (Table 2).

**Table 2:** Impact of dry aging (ND) and wet aging (NW) on physicochemical properties and freshness of fish fillets.

Traits	Aging method	Aging period (day)					Interaction
		0	2	4	6	8	
pH	ND	6.45 ± 0.12^ab^	6.50 ± 0.09^a^	6.51 ± 0.09^a^	6.18 ± 0.13^bB^	6.48 ± 0.12^a^	*F* (4, 20) = 3.648, *p* = 0.0218
	NW	6.45 ± 0.12	6.58 ± 0.08	6.52 ± 0.05	6.53 ± 0.01^A^	6.39 ± 0.18	

TBARS (mg MDA/kg)^1^	ND	0.70 ± 0.13	0.98 ± 0.24	1.27 ± 0.34	1.51 ± 0.53	1.63 ± 0.83	*F* (4, 20) = 0.7632, *p* = 0.5615
	NW	0.70 ± 0.13	0.96 ± 0.16	0.88 ± 0.05	1.03 ± 0.18	1.08 ± 0.31	

VBN (mg/100 g)^2^	ND	7.68 ± 0.95	10.95 ± 1.88	16.58 ± 6.60	15.62 ± 2.95^A^	14.94 ± 4.21	*F* (4, 20) = 0.5854, *p* = 0.6769
	NW	7.68 ± 0.95	9.21 ± 2.55	15.22 ± 8.27	9.31 ± 0.84^B^	11.26 ± 1.76	

Total plate count (log CFU/g)	ND	3.03 ± 0.18^b^	3.37 ± 0.14^ab^	3.41 ± 0.08^ab^	3.71 ± 0.24^a^	3.41 ± 0.36^ab^	*F* (4, 20) = 0.2507, *p* = 0.9058
	NW	3.03 ± 0.18	3.15 ± 0.22	3.43 ± 0.24	3.73 ± 0.82	3.57 ± 0.27	

K‐value (%)	ND	11.83 ± 2.44	11.63 ± 0.75^A^	11.73 ± 1.26^B^	12.16 ± 3.63^B^	13.73 ± 1.07^B^	*F* (4, 20) = 9.066, *p* = 0.0002
	NW	11.83 ± 2.44^b^	10.11 ± 0.57^bB^	20.01 ± 4.04^aA^	23.92 ± 1.97^aA^	20.77 ± 2.03^aA^	

CIE *L* ^∗^^3^	ND	59.82 ± 1.78	55.29 ± 2.15^B^	60.87 ± 2.08	57.11 ± 1.67	56.10 ± 3.04	*F* (4, 20) = 5.284, *p* = 0.0045
	NW	59.82 ± 1.78	64.89 ± 2.45^A^	59.86 ± 2.73	59.99 ± 2.70	61.27 ± 1.93	

CIE *a* ^∗^^4^	ND	−10.44 ± 0.86	−8.96 ± 1.07^A^	−11.85 ± 0.90^B^	−10.03 ± 3.12	−8.72 ± 1.33	*F* (4, 20) = 2.878, *p* = 0.0494
	NW	−10.44 ± 0.86	−12.24 ± 0.23^B^	−9.75 ± 1.83^A^	−10.97 ± 0.46	−10.56 ± 1.65	

CIE *b* ^∗^^5^	ND	17.19 ± 1.83	17.04 ± 2.72	14.62 ± 0.59	18.59 ± 1.23	16.57 ± 3.13	*F* (4, 20) = 0.6927, *p* = 0.6057
	NW	17.19 ± 1.83	17.32 ± 0.55	16.54 ± 1.27	16.85 ± 2.03	17.00 ± 2.24	

Δ*E* ^6^	ND	—	5.49 ± 1.43	3.64 ± 0.30	3.87 ± 2.40	5.29 ± 2.18	*F* (3, 16) = 0.4990, *p* = 0.6882
	NW		5.42 ± 2.36	2.89 ± 0.47	2.65 ± 1.27	2.97 ± 1.19	

*Note:* Data are expressed as means ± SD (*n* = 3). ^a,c^Different letters within the same row indicate significant differences, as determined by one‐way ANOVA with Tukey’s multiple comparisons test (*p* < 0.05). ^A,B^Different letters in the same column indicate significant differences (*p* < 0.05) between aging methods on the same day, as determined by Student’s *t*‐test. The interaction effects were evaluated using a two‐way ANOVA. The absence of superscript letters indicates no significant differences (*p* > 0.05) as determined by one‐way ANOVA or Student’s *t*‐test.

^1^Thiobarbituric acid reactive substances (TBARS) (mg MDA/ kg).

^2^Volatile basic nitrogen (VBN).

^3^ *L* ^∗^, lightness.

^4^ *a* ^∗^, redness.

^5^ *b* ^∗^, yellowness.

^6^Δ*E*, color difference coefficient.

The CIELAB color space was used to evaluate color changes in fish fillets during aging. In dry‐aged fillets, lightness (CIELAB *L* ^∗^) decreased from 59.82 on day 0 to 56.10 on day 8. The redness (*a* ^∗^) value increased to −8.96 on day 2, then declined to −11.85 on day 4, and subsequently rose again to −8.72 by day 8. The yellowness (*b* ^∗^) value dropped to 14.62 on day 4 but increased to 16.57 by day 8.

Wet‐aged fillets exhibited a different pattern. The *L* ^∗^ value increased to 64.89 on day 2 and then declined to 61.27 by day 8. However, the lightness remained significantly higher than that of dry‐aged fillets throughout the aging period (*p* < 0.05). The *a* ^∗^ value decreased to −12.24 on day 2 and then increased to −10.56 by day 8. However, no consistent trend in *b* ^∗^ values was observed during wet aging (Table 2).

To further assess color variations during aging, color differences (Δ*E*) were calculated relative to day 0. The Δ*E* of dry‐aged fillets initially decreased, reaching a minimum of 3.64 on day 4, then gradually increased. Similarly, wet‐aged fillets showed a decline in Δ*E* over time, reaching the lowest value (2.65) on day 6, followed by an increase.

### 3.3. Effects of Dry and Wet Aging on WHC and Cooking Loss of Grouper Fillets

WHC showed opposite trends between the two aging methods. The WHC of dry‐aged fish fillets gradually increased from 98.05% on day 0 to 99.23% by day 8 (*p* < 0.05), indicating improved moisture retention during aging. In contrast, the WHC of wet‐aged fillets decreased from 98.05% to 96.52% over the same period (*p* < 0.05). Cooking loss in dry‐aged fillets increased initially, peaking at 18.70% on day 2, then declined. Meanwhile, the cooking loss of wet‐aged fish fillets increased steadily, reaching 30.77% after 6 days, significantly higher than on day 0 (*p* < 0.05) (Table 3). These findings show that the aging method affects the physicochemical properties of fish fillets. Specifically, dry aging may cause structural changes that boost WHC and reduce moisture loss during cooking. Conversely, wet aging hampers water retention, resulting in higher cooking loss during heat processing.

**Table 3:** Changes in cooking loss and water‐holding capacity during dry aging (ND) and wet aging (NW).

Traits	Aging method	Aging period (day)					Interaction
		0	2	4	6	8	
Water holding capacity (%)	ND	98.05 ± 0.22^b^	98.34 ± 0.22^bA^	98.69 ± 0.12^abA^	98.26 ± 0.50^b^	99.23 ± 0.14^aA^	*F* (4, 20) = 17.57, *p* < 0.0001
	NW	98.05 ± 0.22^a^	97.69 ± 0.15^abB^	96.79 ± 0.19^bcB^	97.28 ± 0.55^ac^	96.52 ± 0.43^cB^	

Cooking loss (%)	ND	8.60 ± 3.13^b^	18.70 ± 3.70^aA^	11.37 ± 1.67^abA^	16.16 ± 2.71^abA^	10.83 ± 4.51^abA^	*F* (4, 20) = 7.957, *p* = 0.0005
	NW	8.60 ± 3.13^c^	28.22 ± 2.42^abB^	23.63 ± 2.75^bB^	30.77 ± 1.09^aB^	27.80 ± 1.78^abB^	

*Note:* Data are expressed as means ± SD (*n* = 3). ^a,c^Different letters within the same row indicate significant differences, as determined by one‐way ANOVA with Tukey’s multiple comparisons test (*p* < 0.05). ^A,B^Different letters in the same column indicate significant differences (*p* < 0.05) between aging methods on the same day, as determined by Student’s *t*‐test. The interaction effects were evaluated using a two‐way ANOVA. The absence of superscript letters indicates no significant differences (*p* > 0.05) as determined by one‐way ANOVA or Student’s *t*‐test.

### 3.4. Texture Profile Changes in Grouper Fillets During Dry and Wet Aging

TPA was conducted to assess the effects of aging on the textural properties of fish fillets. In dry‐aged fillets, all texture parameters exhibited fluctuating, nonlinear patterns throughout aging. Hardness reached its lowest value (9.65 N) on day 4, after which it gradually increased. Cohesiveness and springiness peaked on day 6 at 0.71 cm and 0.64 cm, respectively, with cohesiveness showing a significant (*p* < 0.05) increase compared to day 0. Similarly, gumminess and chewiness attained their highest values on day 6, reaching 6.55 N and 21.36 N·cm, respectively. Chewiness was also significantly (*p* < 0.05) elevated compared to day 0. By contrast, wet‐aged fillets showed a more consistent increase in hardness, peaking at 13.73 N on day 6, significantly (*p* < 0.01) higher than on day 0. Cohesiveness decreased to 0.41 on day 4 and recovered slightly to 0.48 by day 8. Springiness peaked at 0.60 cm on day 4. Gumminess and chewiness increased during the early phase of aging, then decreased to 4.83 N and 15.55 N·cm, respectively, on day 4, and subsequently rose again by day 8 (Table 4).

**Table 4:** Texture properties of grouper fillets during dry aging (ND) and wet aging (NW).

Parameters	Aging method	Aging period (day)					Interaction
		0	2	4	6	8	
Hardness (N)	ND	6.29 ± 1.16	15.41 ± 1.83 ^∗∗∗^	9.65 ± 0.99	10.35 ± 2.07 ^∗^	10.08 ± 2.01	*F* (4, 20) = 3.304, *p* = 0.0312
	NW		11.83 ± 0.17 ^∗^	10.43 ± 3.07	13.73 ± 2.50 ^∗∗^	12.26 ± 0.92 ^∗^	

Cohesiveness	ND	0.38 ± 0.15	0.51 ± 0.01	0.49 ± 0.03	0.71 ± 0.23 ^∗^	0.50 ± 0.01	*F* (4, 20) = 1.982, *p* = 0.1362
	NW		0.45 ± 0.07	0.41 ± 0.04	0.41 ± 0.04	0.48 ± 0.05	

Springiness (cm)	ND	0.53 ± 0.06	0.58 ± 0.08	0.57 ± 0.01	0.64 ± 0.04	0.56 ± 0.00	*F* (4, 20) = 1.611, *p* = 0.2106
	NW		0.58 ± 0.07	0.60 ± 0.02	0.52 ± 0.03	0.54 ± 0.07	

Gumminess (N)	ND	2.45 ± 1.18	7.80 ± 0.93 ^∗∗∗^	4.84 ± 0.51	6.55 ± 1.46 ^∗∗^	5.01 ± 1.25	*F* (4, 20) = 1.735, *p* = 0.1819
	NW		5.21 ± 0.48	4.83 ± 2.05	5.82 ± 1.60 ^∗^	6.09 ± 1.19 ^∗^	

Chewiness (N 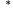 cm)	ND	6.80 ± 3.73	23.41 ± 4.53 ^∗∗^	14.35 ± 1.91	21.36 ± 5.65 ^∗∗^	14.04 ± 3.95	*F* (4, 20) = 1.516, *p* = 0.2355
	NW		15.35 ± 2.60	15.55 ± 8.16	16.70 ± 3.55	17.03 ± 4.34	

*Note:* Data are expressed as means ± SD (*n* = 3). Statistical analysis within the same row was performed using one‐way ANOVA, followed by Dunnett’s multiple comparison test, comparing each time point to day 0.  ^∗^ means *p* < 0.05,  ^∗∗^ means *p* < 0.01, and  ^∗∗∗^ means *p* < 0.001. The interaction effects were evaluated using a two‐way ANOVA. The absence of superscript letters indicates no significant differences (*p* > 0.05) as determined by one‐way ANOVA or Student’s *t*‐test.

From a textural perspective, these findings suggest that day 4 represents a textural balance aging point for both dry‐ and wet‐aged grouper fillets. At this time, dry‐aged fillets exhibited the lowest hardness, while other parameters peaked on day 6, indicating a firmer muscle structure that requires greater chewing force. On the other hand, in wet‐aged fillets, hardness peaked later (on day 6); however, except for springiness, most texture parameters declined on day 4, suggesting that wet‐aged fillets at this stage possessed desirable tenderness and chewiness. Moreover, on day 4, no significant differences were observed between dry‐ and wet‐aged samples across all measured texture parameters.

### 3.5. Changes in Fish Muscle Proteins During Aging

The contents of TCA‐soluble peptides were measured to assess protein breakdown during aging. Results showed an increase in TCA‐soluble peptides over time in dry‐aged fish fillets, indicating continuous proteolytic activity. In contrast, the wet‐aged fish fillets showed a fluctuating trend: The TCA‐soluble peptide content decreased to 1.29 mg/g on day 4, then increased to 1.56 mg/g on day 6, and finally declined again to 1.32 mg/g by day 8 (Figure 2a). SDS‐PAGE analysis revealed that both dry‐ and wet‐aged fish fillets experienced changes in myofibrillar protein composition during aging. These changes involved several key proteins, including myosin heavy chain (MHC, 220 kDa), α‐actinin (100 kDa), actin (43 kDa), troponin T (36 kDa), and tropomyosin (35 kDa) (Figure 2b). Quantitative analysis of SDS‐PAGE bands related to myofibrillar proteins showed that in dry‐aged fish fillets, the MHC band intensity decreased over time. In contrast, the wet‐aged fish fillets have the band intensity. On day 8, the wet‐aged fillets had significantly (*p* < 0.05) higher MHC band intensity than the dry‐aged fillets (Figure 2c). A slight decrease in tropomyosin was also observed in the dry‐aged fillets. Conversely, wet‐aged fillets showed a significant (*p* < 0.05) increase in tropomyosin levels on day 8 (Figure 2d).

Figure 2Changes in fish muscle proteins during aging. (a) Changes in trichloroacetic acid (TCA)–soluble peptide contents in dry‐aging and wet‐aging grouper fillets. (b) SDS‐PAGE analysis of protein extracts from grouper fillets during dry‐aging and wet‐aging. Densitometric quantification of (c) myosin heavy chain (MHC) and (d) tropomyosin. Data are presented as mean ± SD (*n* = 3). For comparisons between different aging methods at the same time point, an independent Student’s *t*‐test was conducted.  ^∗^ *p* < 0.05.

**Figure:**
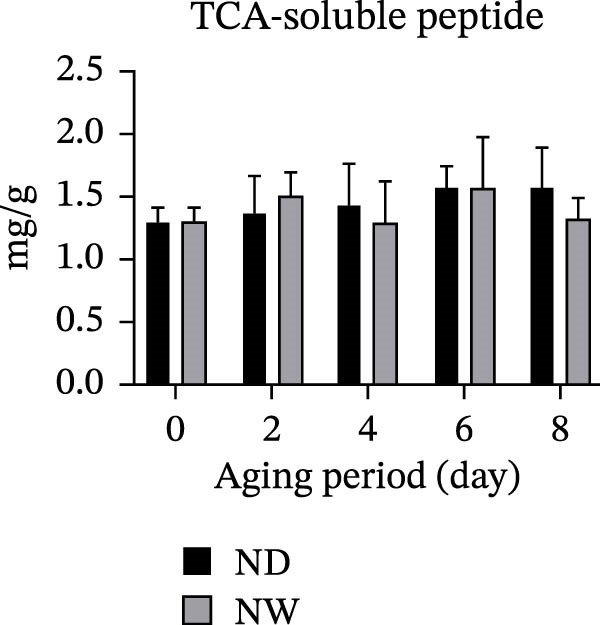
(a)

**Figure:**
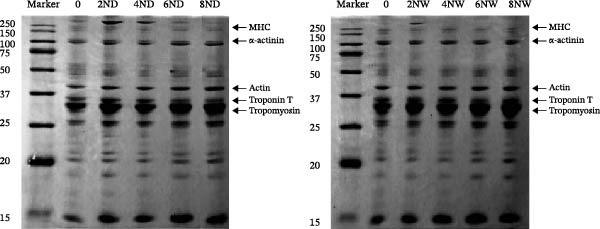
(b)

**Figure:**
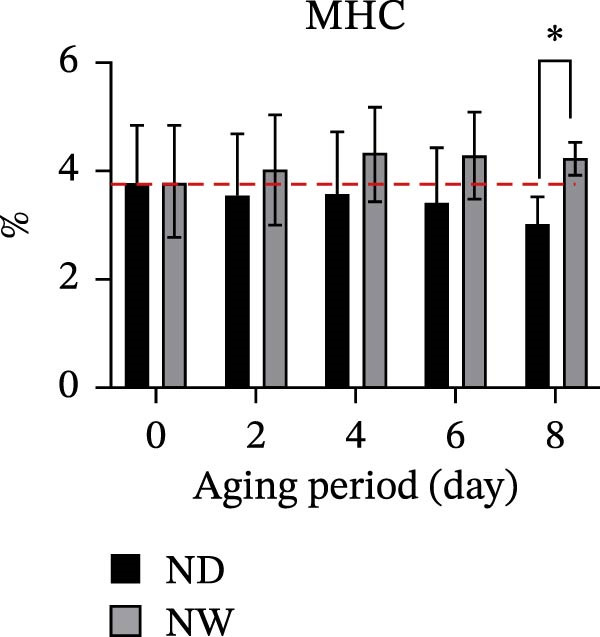
(c)

**Figure:**
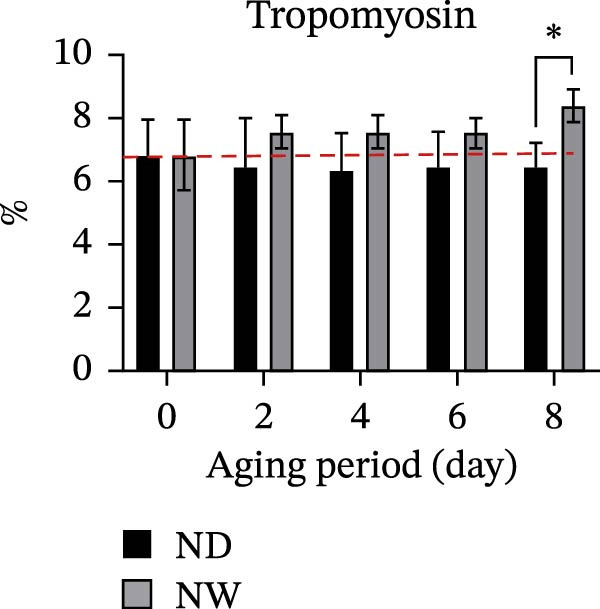
(d)

### 3.6. Changes in FAAs and Nucleotides and TAVs During Dry and Wet Aging of Grouper Fillets

The total FAA content varied over time, depending on the aging method. The FAA concentration in dry‐aged fish fillets gradually increased during aging, reaching its peak at 931.38 mg/100 g on day 4 (Table 5). In contrast, the wet‐aged fish fillets reached their maximum FAA content of 694.59 mg/100 g earlier, on day 2 (Table 5). Umami‐related amino acids showed different trends throughout the aging process. Glutamic acid and aspartic acid in dry‐aged fish fillets peaked on day 4 at 64.29 and 35.63 mg/100 g (Table 5), respectively, before declining. Conversely, glutamic acid and aspartic acid in wet‐aged fish fillets steadily increased during aging, reaching 51.92 and 29.12 mg/100 g, respectively, on day 8 (Table 5). Among sweet‐tasting amino acids, glycine peaked in the dry‐aged fish fillets on day 4 (181.92 mg/100 g) (Table 5). In wet‐aged fish fillets, glycine, serine, and alanine peaked early, on day 2 (Table 5). The bittersweet amino acid arginine increased consistently during dry aging, reaching 315.43 mg/100 g on day 8, significantly (*p* < 0.05) higher than in wet‐aged fish fillets (Table 5). Lysine followed a similar trend in both aging processes; it peaked on day 4 at 55.25 mg/100 g in dry‐aged and 42.68 mg/100 g in wet‐aged fish fillets, then declined. Proline showed aging‐related variations. In dry‐aged fish fillets, proline peaked at 52.26 mg/100 g on day 8. In wet‐aged fillets, proline increased gradually, peaking on day 8 at 26.34 mg/100 g (Table 5).

**Table 5:** Changes in the contents of free amino acids in grouper fillets during dry aging (ND) and wet aging (NW).

FAA (mg/100 g on wet weight)	Taste attribute	Aging method	Aging time (day)					Interaction
			0	2	4	6	8	
Aspartic acid	Umami	ND	23.26 ± 10.41	25.38 ± 2.66	35.63 ± 9.70	15.86 ± 3.62	29.96 ± 11.61	*F* (4, 20) = 0.5101, *p* = 0.7290
		NW		21.05 ± 4.40	27.34 ± 4.15	19.87 ± 3.79	29.12 ± 10.88	

Glutamic acid	Umami	ND	29.23 ± 13.04	42.48 ± 9.98	64.29 ± 21.90	33.89 ± 18.43	64.04 ± 14.71	*F* (4, 20) = 0.7783, *p* = 0.5523
		NW		44.45 ± 13.96	49.84 ± 1.82	42.05 ± 3.49	51.92 ± 10.41	

Serine	Sweet	ND	21.33 ± 9.30	31.38 ± 8.27	46.20 ± 9.57^A^	24.30 ± 12.69	110.59 ± 113.72	*F* (4, 20) = 1.238, *p* = 0.3266
		NW		38.87 ± 8.21	28.87 ± 2.24^B^	24.13 ± 4.45	35.63 ± 14.44	

Glycine	Sweet	ND	70.01 ± 49.42	119.88 ± 8.28	181.92 ± 32.18	131.03 ± 104.07	159.14 ± 13.74	*F* (4, 20) = 0.8205, *p* = 0.5273
		NW		147.49 ± 24.38	127.10 ± 40.44	136.53 ± 24.14	121.82 ± 26.78	

Arginine	Bitter/sweet	ND	188.94 ± 133.56	292.04 ± 87.16	418.92 ± 138.59	247.14 ± 184.70	315.43 ± 229.30^A^	*F* (4, 20) = 0.4781, *p* = 0.7514
		NW		306.61 ± 31.52	289.45 ± 71.33	311.02 ± 33.30	301.50 ± 46.20^B^	

Alanine	Sweet	ND	37.11 ± 23.24	66.05 ± 20.11	99.01 ± 37.12	56.33 ± 40.36	36.47 ± 36.20	*F* (4, 20) = 1.271, *p* = 0.3143
		NW		78.86 ± 22.29	66.61 ± 16.72	62.49 ± 10.06	69.99 ± 9.47	

Proline	Sweet/bitter	ND	16.77 ± 6.06	20.53 ± 3.60	31.01 ± 7.41	18.38 ± 7.98	52.26 ± 50.22	*F* (4, 20) = 0.6399, *p* = 0.6402
		NW		22.10 ± 6.21	21.75 ± 3.46	15.64 ± 3.91	26.34 ± 12.50	

Lysine	Sweet/bitter	ND	22.86 ± 9.20^b^	34.04 ± 5.47	55.25 ± 21.52	30.42 ± 13.84	38.44 ± 4.23	*F* (4, 20) = 0.8703, *p* = 0.4989
		NW		35.15 ± 5.00^ab^	42.68 ± 5.17^a^	38.94 ± 6.54^ab^	40.77 ± 7.50^ab^	

Total FAA	—	ND	409.51 ± 227.41	631.77 ± 89.39	931.38 ± 272.95	557.35 ± 384.96	806.34 ± 104.52	*F* (4, 20) = 0.9318, *p* = 0.4655
		NW		694.59 ± 98.55	653.64 ± 122.94	650.67 ± 71.38	677.10 ± 54.65	

*Note:* Data are expressed as means ± SD (*n* = 3). The interaction effects were evaluated using a two‐way ANOVA. The absence of superscript letters indicates no significant differences (*p* > 0.05) as determined by one‐way ANOVA or Student’s *t*‐test. ^a,b^Different letters within the same row indicate significant differences, as determined by one‐way ANOVA with Tukey’s multiple comparisons test (*p* < 0.05). ^A,B^Different letters in the same column indicate significant differences (*p* < 0.05) between aging methods on the same day, as determined by Student’s *t*‐test.

The degradation patterns of nucleotides in fish fillets subjected to both aging methods showed notable differences. In dry‐aged fish fillets, flavor‐related nucleotides ATP, ADP, and AMP reached their highest concentrations early, on day 2, measuring 16.37 μmol/L, 45.14 μmol/L, and 58.72 μmol/L, respectively, before steadily decreasing during the remaining aging period. The umami‐associated nucleotide IMP also peaked on day 2 at 1444.92 μmol/L before gradually declining. Conversely, the breakdown of inosine and hypoxanthine accelerated with aging time. Inosine reached its maximum (259.94 μmol/L) on day 8, while hypoxanthine peaked earlier, on day 2, at 359.67 μmol/L. In wet‐aged fish fillets, ATP, ADP, AMP, and IMP showed a continuous decline over the 8‐day aging period. Meanwhile, inosine levels steadily increased to 122.41 μmol/L by day 8, and hypoxanthine reached its highest concentration (434.65 μmol/L) on day 6, significantly (*p* < 0.05) higher than in dry‐aged fish fillets. Overall, dry aging preserved higher initial levels of flavor‐enhancing nucleotides such as IMP but also resulted in a more rapid accumulation of degradation products, including inosine and hypoxanthine. In contrast, wet aging resulted in a more gradual decline of nucleotides, with lower peak levels of umami compounds and a delayed increase in bitter‐tasting degradation products (Table 6).

**Table 6:** Changes in the contents of nucleotide in grouper fillets during dry aging (ND) and wet aging (NW).

Nucleotide (umol/L on wet weight)	Taste attribute	Aging method	Aging time (day)					Interaction
			0	2	4	6	8	
ATP	—	ND	15.28 ± 7.07	16.37 ± 7.77	14.09 ± 3.01	12.61 ± 2.97	12.72 ± 7.68	*F* (4, 20) = 0.1162, *p* = 0.9752
		NW		12.86 ± 7.93	10.36 ± 3.55	9.56 ± 2.27	9.06 ± 2.24	

ADP	—	ND	43.98 ± 9.45	45.14 ± 5.05	42.27 ± 9.38	37.89 ± 11.31	36.72 ± 22.22	*F* (4, 20) = 0.1914, *p* = 0.9401
		NW		37.57 ± 23.25	29.34 ± 9.52	31.67 ± 8.56	30.02 ± 6.34	

AMP	Umami/sweet	ND	43.67 ± 11.51	58.72 ± 6.51	45.83 ± 8.44	51.51 ± 13.08	51.59 ± 26.53	*F* (4, 20) = 0.4944, *p* = 0.7400
		NW		37.81 ± 18.51	34.29 ± 10.90	34.98 ± 10.10	35.90 ± 10.24	

IMP	Umami	ND	1082.36 ± 295.50	1444.92 ± 261.74	1125.09 ± 304.29	1305.05 ± 468.58	1192.96 ± 747.59	*F* (4, 20) = 0.4386, *p* = 0.7792
		NW		990.74 ± 685.66	681.43 ± 264.14	701.34 ± 252.47	733.82 ± 271.78	

Inosine	—	ND	78.00 ± 9.88	122.99 ± 63.88	202.62 ± 56.39	200.18 ± 66.14	259.94 ± 125.23	*F* (4, 20) = 1.121, *p* = 0.3744
		NW		84.53 ± 69.39	115.74 ± 23.89	115.66 ± 60.74	122.41 ± 5.62	

Hypoxanthine	Bitter	ND	279.62 ± 19.40	359.67 ± 180.61	161.90 ± 47.65	212.49 ± 45.33^B^	189.83 ± 124.43	*F* (4, 20) = 3.019, *p* = 0.0423
		NW		200.26 ± 128.93	302.75 ± 128.66	434.65 ± 124.89^A^	351.67 ± 122.16	

*Note:* Data are expressed as means ± SD (*n* = 3). The interaction effects were evaluated using a two‐way ANOVA. The absence of superscript letters indicates no significant differences (*p* > 0.05) as determined by one‐way ANOVA or Student’s *t*‐test. ^A,B^Different letters in the same column indicate significant differences (*p* < 0.05) between aging methods on the same day, as determined by Student’s *t*‐test.

TAVs were calculated (Table 7) to assess the flavor contribution of these compounds. In dry‐aged fish fillets, glutamic acid, arginine, and IMP exhibited TAV increases by day 4. Alanine and glycine increased from initial TAVs of 0.62 and 0.54 to 1.65 and 1.40, respectively, reaching perceptible thresholds. Additionally, lysine reached a TAV of 1.11 on day 4. On the other hand, in wet‐aged fish fillets, glutamic acid and IMP increased by day 2, with arginine, alanine, and glycine also exceeding the threshold of one.

**Table 7:** Taste activity values (TAVs) of amino acids and their corresponding flavor characteristics in grouper fillets during dry aging (ND) and wet aging (NW).

TAV of FAA	Taste attribute	Taste threshold (mg/100 mL)^1,2^	Aging method	Aging time (day)			Interaction
				0	2	4	
Aspartic acid	Umami	100	ND	0.23 ± 0.10	0.25 ± 0.03	0.36 ± 0.10	*F* (2, 12) = 0.4361, *p* = 0.6564
			NW		0.21 ± 0.04	0.27 ± 0.04	

Glutamic acid	Umami	30	ND	0.97 ± 0.43	1.42 ± 0.33	2.14 ± 0.73	*F* (2, 12) = 0.6477, *p* = 0.5406
			NW		1.48 ± 0.47	1.66 ± 0.06	

Serine	Sweet	150	ND	0.14 ± 0.06^b^	0.21 ± 0.06^ab^	0.31 ± 0.06^aA^	*F* (2, 12) = 0.3602, *p* = 0.0595
			NW		0.26 ± 0.05	0.19 ± 0.01^B^	

Glycine	Sweet	130	ND	0.54 ± 0.38^b^	0.92 ± 0.06^ab^	1.40 ± 0.25^a^	*F* (2, 12) = 0.1928, *p* = 0.1879
			NW		1.13 ± 0.19	0.98 ± 0.31	

Arginine	Bitter/sweet	50	ND	3.78 ± 2.67	5.84 ± 1.74	8.36 ± 2.77	*F* (2, 12) = 0.8151, *p* = 0.4657
			NW		6.13 ± 0.63	5.79 ± 1.43	

Alanine	Sweet	60	ND	0.62 ± 0.39	1.10 ± 0.34	1.65 ± 0.62	*F* (2, 12) = 0.1343, *p* = 0.2977
			NW		1.31 ± 0.37	1.11 ± 0.28	

Proline	Sweet/bitter	300	ND	0.06 ± 0.02	0.07 ± 0.01	0.10 ± 0.02	*F* (2, 12) = 0.1608, *p* = 0.2406
			NW		0.07 ± 0.02	0.07 ± 0.01	

Lysine	Sweet/bitter	50	ND	0.46 ± 0.18^b^	0.68 ± 0.11	1.11 ± 0.43	*F* (2, 12) = 0.7286, *p* = 0.5027
			NW		0.70 ± 0.10^ab^	0.85 ± 0.10^a^	

AMP	Umami/sweet	50	ND	0.03 ± 0.01	0.04 ± 0.01	0.03 ± 0.00	*F* (2, 12) = 1.175, *p* = 0.3419
			NW		0.03 ± 0.01	0.02 ± 0.01	

IMP	Umami	25	ND	1.51 ± 0.41	2.01 ± 0.36	1.57 ± 0.40	*F* (2, 12) = 0.6908, *p* = 0.5201
			NW		1.38 ± 0.96	0.95 ± 0.37	

*Note:* Data are expressed as means ± SD (*n* = 3). ^a,b^Different letters within the same row indicate significant differences, as determined by one‐way ANOVA with Tukey’s multiple comparisons test (*p* < 0.05). ^A,B^Different letters in the same column indicate significant differences (*p* < 0.05) between aging methods on the same day, as determined by Student’s *t*‐test. The interaction effects were evaluated using a two‐way ANOVA. The absence of superscript letters indicates no significant differences (*p* > 0.05) as determined by one‐way ANOVA or Student’s *t*‐test.

^1^(Gunlu and Gunlu [17]).

^2^(Zhang et al. [23]).

### 3.7. Changes in Fatty Acid Composition

To further examine how aging affects the lipid nutritional quality of grouper fillets, the changes in fatty acid profiles were analyzed (Table 8). The main fatty acids identified included palmitic acid, oleic acid, linoleic acid, and docosahexaenoic acid (DHA). Palmitic acid remained the most abundant saturated fatty acid (SFA). In the ND group, it decreased from an initial value of 0.679 ± 0.346 g/100 g to a minimum of 0.317 ± 0.091 g/100 g on day 4. A similar decline was seen in the NW group, which dropped to 0.487 ± 0.315 g/100 g by day 8. Other SFAs, such as myristic acid and stearic acid, showed no significant change during the process.

**Table 8:** Changes in the contents of fatty acids in grouper fillets during dry aging (ND) and wet aging (NW).

Fatty acids (g/100 g on wet weight)	Aging method	Aging time (day)					Interaction^#^
		0	2	4	6	8	
Myristic acid (C14:0)	ND	0.083 ± 0.051	0.070 ± 0.056	0.033 ± 0.015	0.070 ± 0.044	0.040 ± 0.017	*F* (4, 20) = 0.2151, *p* = 0.9269
	NW		0.070 ± 0.036	0.053 ± 0.021	0.050 ± 0.035	0.047 ± 0.029	

Palmitic acid (C16:0)	ND	0.679 ± 0.346	0.617 ± 0.463	0.317 ± 0.091	0.650 ± 0.404	0.403 ± 0.181	*F* (4, 20) = 0.2098, *p* = 0.9299
	NW		0.593 ± 0.300	0.510 ± 0.195	0.527 ± 0.359	0.487 ± 0.315	

Stearic acid (C18:0)	ND	0.207 ± 0.075	0.183 ± 0.114	0.107 ± 0.023	0.197 ± 0.112	0.130 ± 0.046	*F* (4, 20) = 0.2439, *p* = 0.9099
	NW		0.163 ± 0.076	0.153 ± 0.050	0.163 ± 0.110	0.160 ± 0.095	

Lignoceric acid (C24:0)	ND	0.073 ± 0.040	0.060 ± 0.046	0.033 ± 0.015	0.067 ± 0.040	0.047 ± 0.029	*F* (4, 20) = 0.3921, *p* = 0.8118
	NW		0.073 ± 0.040	0.047 ± 0.023	0.040 ± 0.017	0.037 ± 0.021	

Palmitoleic acid (C16:1)	ND	0.120 ± 0.060	0.103 ± 0.081	0.047 ± 0.015	0.110 ± 0.070	0.060 ± 0.026	*F* (4, 20) = 0.2850, *p* = 0.8842
	NW		0.097 ± 0.051	0.080 ± 0.036	0.080 ± 0.061	0.077 ± 0.055	

Oleic acid (9c‐C18:1)	ND	0.960 ± 0.593	0.803 ± 0.561	0.410 ± 0.183	0.797 ± 0.486	0.557 ± 0.289	*F* (4, 20) = 0.1857, *p* = 0.9431
	NW		0.880 ± 0.469	0.650 ± 0.286	0.610 ± 0.373	0.550 ± 0.339	

Vaccenic acid (11c‐C18:1)	ND	0.103 ± 0.061	0.080 ± 0.060	0.037 ± 0.021	0.087 ± 0.051	0.057 ± 0.029	*F* (4, 20) = 0.2170, *p* = 0.9258
	NW		0.093 ± 0.057	0.067 ± 0.032	0.067 ± 0.046	0.060 ± 0.044	

Linoleic acid (9c, 12c‐C18:2)	ND	0.907 ± 0.702	0.733 ± 0.454	0.377 ± 0.240	0.693 ± 0.377	0.557 ± 0.306	*F* (4, 20) = 0.3777, *p* = 0.8219
	NW		0.917 ± 0.488	0.577 ± 0.300	0.430 ± 0.151	0.340 ± 0.173	

α‐Linolenic acid (9c, 12c, 15c‐C18:3)	ND	0.107 ± 0.083	0.083 ± 0.050	0.043 ± 0.032	0.077 ± 0.042	0.063 ± 0.040	*F* (4, 20) = 0.2867, *p* = 0.8831
	NW		0.113 ± 0.057	0.067 ± 0.035	0.057 ± 0.031	0.047 ± 0.029	

Arachidonic acid (5c, 8c, 11c, 14c‐C20:4)	ND	0.027 ± 0.006	0.027 ± 0.012	0.017 ± 0.006	0.023 ± 0.021	0.020 ± 0.000	*F* (4, 20) = 0.4375, *p* = 0.7800
	NW		0.020 ± 0.010	0.023 ± 0.006	0.017 ± 0.012	0.017 ± 0.012	

Eicosapentaenoic acid (EPA) (5c, 8c, 11c, 14c, 17c‐C20:5)	ND	0.007 ± 0.006	0.003 ± 0.006	0.003 ± 0.006	0.023 ± 0.006	0.013 ± 0.006	*F* (4, 20) = 2.393, *p* = 0.0848
	NW		0.020 ± 0.000	0.020 ± 0.000	0.023 ± 0.006	0.017 ± 0.015	

Docosapentaenoic acid (7c, 10c, 13c, 16c, 19c‐C22:5)	ND	0.037 ± 0.021	0.030 ± 0.020	0.017 ± 0.006	0.033 ± 0.021	0.023 ± 0.006	*F* (4, 20) = 0.2209, *p* = 0.9236
	NW		0.030 ± 0.017	0.023 ± 0.012	0.023 ± 0.015	0.017 ± 0.021	

Docosahexaenoic acid (DHA) (4c, 7c, 10c, 13c, 16c, 19c‐C22:6)	ND	0.140 ± 0.053	0.127 ± 0.076	0.070 ± 0.020	0.127 ± 0.068	0.097 ± 0.038	*F* (4, 20) = 0.2700, *p* = 0.8938
	NW		0.120 ± 0.056	0.097 ± 0.023	0.093 ± 0.049	0.077 ± 0.067	

*Note:* Data are expressed as means ± SD (*n* = 3). The interaction effects were evaluated using a two‐way ANOVA. The absence of superscript letters indicates no significant differences (*p* > 0.05) as determined by one‐way ANOVA or Student’s *t*‐test.

Oleic acid was the predominant monounsaturated fatty acid (MUFA), decreasing from 0.960 ± 0.593 g/100 g to 0.557 ± 0.289 g/100 g in the ND group and 0.550 ± 0.339 g/100 g in the NW group on day 8. Regarding PUFAs, linoleic acid and DHA decreased in both groups. DHA content in the NW group declined from 0.140 ± 0.053 g/100 g at day 0 to 0.077 ± 0.067 g/100 g by day 8.

There were no significant interaction effects between the aging method and aging time for the fatty acid profiles (*p* > 0.05). Additionally, the levels of SFA, MUFA, and PUFA showed no significant difference over the 8‐day aging period in both dry‐ and wet‐aged fillets. Notably, the levels of n‐3 PUFAs, such as eicosapentaenoic acid (EPA) and DHA, remained stable with no significant degradation. These findings indicated that the fatty acid composition of the grouper fillets was preserved during low‐temperature aging.

### 3.8. Changes in the Metabolites of Grouper Fillets During Aging

A nontargeted metabolomics analysis was performed using LC‐MS to examine metabolic changes during fish fillet aging. A total of 2740 metabolites were identified through comparison with the Human Metabolome Database (HMDB). PCA was performed, with PC1 and PC2 accounting for 19.3% and 13.2% of the total variance, respectively. Further statistical analysis using permutational multivariate ANOVA (PERMANOVA) revealed significant differences in metabolite profiles before and after aging, as well as between the two aging methods (*F* = 10.243, *R* ^2^ = 0.80381, and *p* = 0.001). Notably, dry‐aged fillets sampled on days 4 and 8 exhibited similar metabolite profiles (Figure 3a), indicating that metabolic changes tend to slow after the initial aging period.

Figure 3Untargeted metabolomic analysis of dry‐aging and wet‐aging grouper fillets. (a) Principal component analysis (PCA) score plot of metabolites from dry‐aging and wet‐aging grouper fillets. Variable importance in projection (VIP) scores derived from partial least squares discriminant analysis (PLS‐DA) of grouper fillets after 4 days of (b) dry‐aging and (c) wet‐aging. Variables with VIP scores > 1.0 were considered significant contributors to group discrimination. Volcano plot of metabolites from (d) dry‐aging and (e) wet‐aging grouper fillets on day 0 and day 4. In volcano plots, the *x*‐axis represents log2 fold change (FC), and the *y*‐axis represents log10 false discovery rate (FDR)‐adjusted *p*‐values. Metabolites were considered significant at *p* < 0.05 with FC > 2 or FC < 0.5.

**Figure:**
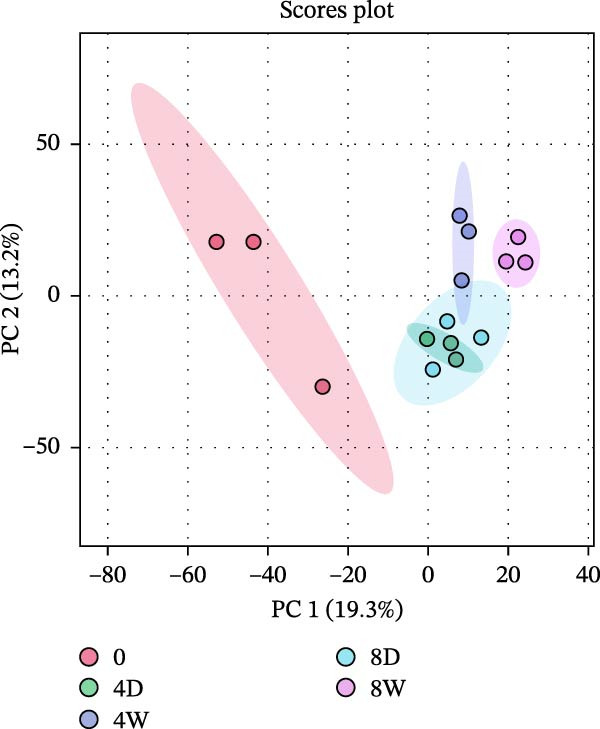
(a)

**Figure:**
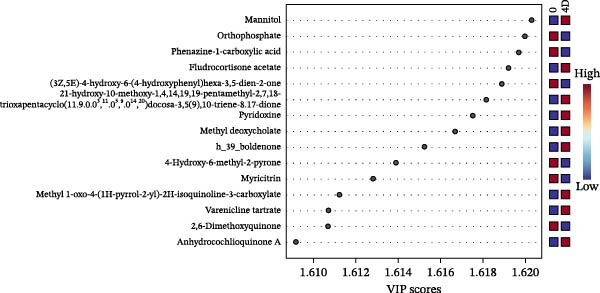
(b)

**Figure:**
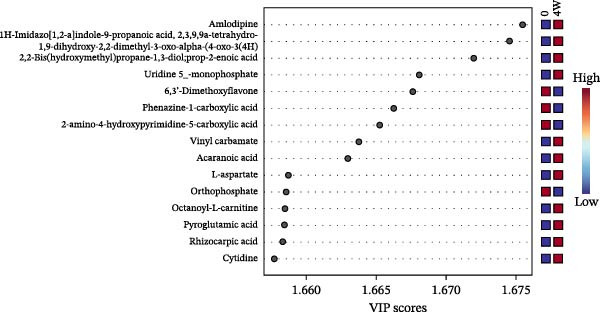
(c)

**Figure:**
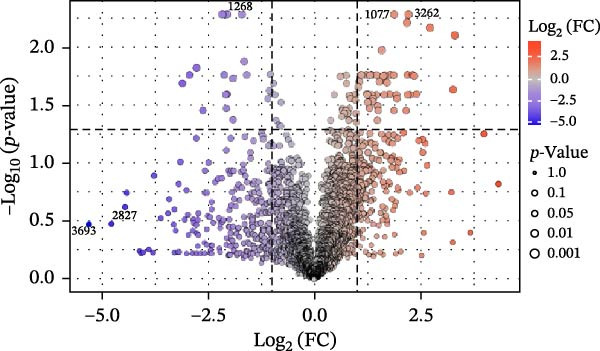
(d)

**Figure:**
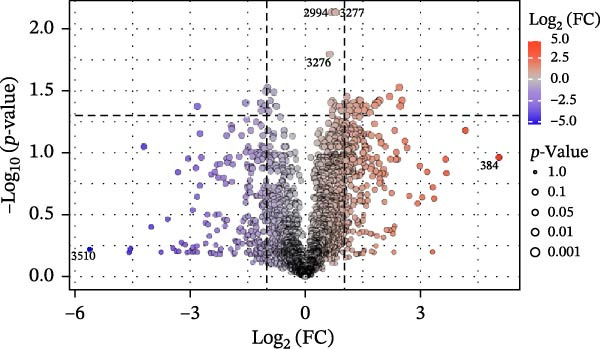
(e)

Partial least squares discriminant analysis (PLS‐DA) was used to identify key metabolites that differentiate the groups. Metabolites with VIP scores above one were considered significant contributors. By day 4, high VIP scores in dry‐aged fish fillets included mannitol, pyridoxine (the alcohol form of vitamin B6), and anhydrocochlioquinone A, all of which are linked to microbial activity and biochemical changes during aging (Figure 3b). Meanwhile, in wet‐aged fish fillets, several metabolites involved in microbial activity, protein hydrolysis, or flavor development, such as uridine 5^′^‐monophosphate (UMP), L‐aspartic acid, pyroglutamic acid, and cytidine, showed elevated VIP scores by day 4 (Figure 3c).

Additionally, *p* < 0.05 and fold change (FC) thresholds > 2 or <0.5 were used to identify metabolites with significant changes. Compared with day 0, metabolites such as pyridoxine, L‐asparagine, inosine, and histamine showed increased levels after 4 days of dry aging (Figure 3d and Table 9). In contrast, metabolites, including carnitine and myricitrin, exhibited decreased levels (Figure 3d and Table 9). Conversely, after 4 days of wet aging, metabolites, including L‐aspartate, histamine, and octanoyl‐L‐carnitine, showed increased levels (Figure 3e and Table 10). However, seven metabolites, including myricitrin, had lower levels (Figure 3e and Table 10). These findings demonstrated that both aging methods produce distinct metabolite expression profiles.

**Table 9:** Impact of dry‐aging method on flavor‐related metabolic differences in the grouper fillets.

Ion (m/z)	Metabolite name	Fold change (ND day 4/day 0)	Peak intensity (ND day 0)	Peak intensity (ND day 4)	Log10 (FDR) adjusted *p*‐values
170.0607	Pyridoxine	6.6204	41,910.11	277,460	0.006478
515.3004	Anhydrocochlioquinone A	4.5923	478,686.4	2,198,285	0.016665
133.05635	L‐asparagine	3.6695	31,029	113,860.8	0.016761
269.09448	Inosine	3.1395	2,055,007	6,451,611	0.024741
205.0708	Mannitol	2.9036	30,190.11	87,658.89	0.019127
112.08177	Histamine	2.4709	125,185.7	309,320.7	0.032634
162.11475	Carnitine	0.4713	118,673.6	55,931.33	0.02682
503.06525	Myricitrin	0.14719	75,999	11,186	0.014447

*Note:* Metabolites were screened based on *p* < 0.05 and the threshold criteria of FC > 2 or FC < 0.5.

**Table 10:** Impact of wet‐aging method on flavor‐related metabolic differences in the grouper fillets.

Ion (m/z)	Metabolite name	Fold change (NW day 4/day 0)	Peak intensity (NW day 0)	Peak intensity (NW day 4)	Log10 (FDR) adjusted *p*‐values
134.04948	L‐aspartate	5.792	71,138.78	412,036.9	0.037799
325.1276	Uridine 5‐monophosphate	5.5151	5479.889	30,222.11	0.02961
112.08177	Histamine	2.2473	125,185.7	281,324.4	0.042245
333.14877	Sinapine	2.5916	24,879.11	64,477.22	0.045591
288.21674	Octanoyl‐L‐carnitine	3.8148	8235.667	31,417.67	0.037799
503.06525	Myricitrin	0.34315	75,999	26,079.33	0.039576

*Note:* Metabolites were screened based on *p* < 0.05 and the threshold criteria of FC > 2 or FC < 0.5.

### 3.9. Correlation Between Quality and Flavor Attributes and Metabolite Profiles in Aged Grouper Fillets

A correlation heatmap was constructed to explore the relationship between metabolite profiles and the quality and flavor features of aged fish fillets. Selected quality and freshness indicators included pH, TPC, VBN, and K‐value. Indicators of oxidative stability and flavor, such as TBARS, FAA, glutamic acid, IMP, and arginine (based on high TAV values), were also included. Metabolites were selected based on the top 15 VIP scores and significant changes seen in volcano plot analysis (FC > 2 or <0.5), especially those related to microbial metabolism, nucleotide turnover, amino acid catabolism, and lipid metabolism.

The Spearman correlation heatmap showed significant associations between key metabolites and quality parameters in dry‐aged fish fillets. Mannitol, pyridoxine, and anhydrocochlioquinone A were strongly and positively correlated with glutamic acid, IMP, and total FAA (*p* < 0.05 to *p* < 0.001), indicating a potential role in flavor improvement during dry aging. Conversely, myricitrin and carnitine showed significant negative correlations with TBARS and K‐value (*p* < 0.05), suggesting antioxidant potential. Histamine and inosine showed positive correlations with IMP, TBARS, and TPC (*p* < 0.05), hinting at involvement in microbial degradation and nucleotide breakdown. These findings emphasized the dual role of metabolites in flavor development and spoilage control (Figure 4a).

Figure 4Correlation between quality and flavor attributes and metabolite profiles in aged grouper fillets. (a) Dry‐aging and (b) wet‐aging grouper fillets. Red and blue colors indicate positive and negative correlation coefficients, respectively. Statistical significance is indicated as follows:  ^∗^ *p* < 0.05,  ^∗∗^ *p* < 0.01,  ^∗∗∗^ *p* < 0.001, and  ^∗∗∗∗^ *p* < 0.0001.

**Figure:**
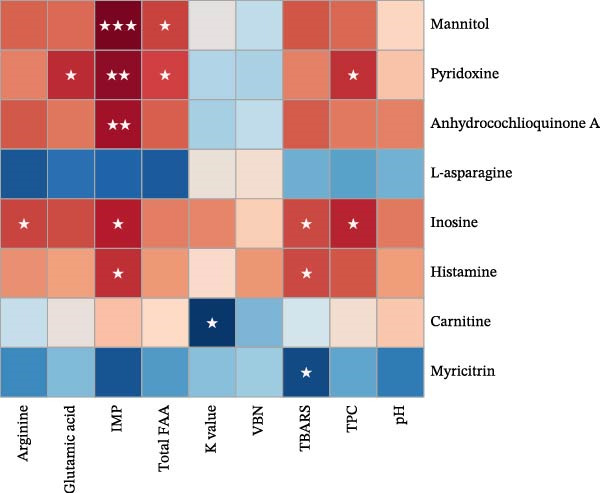
(a)

**Figure:**
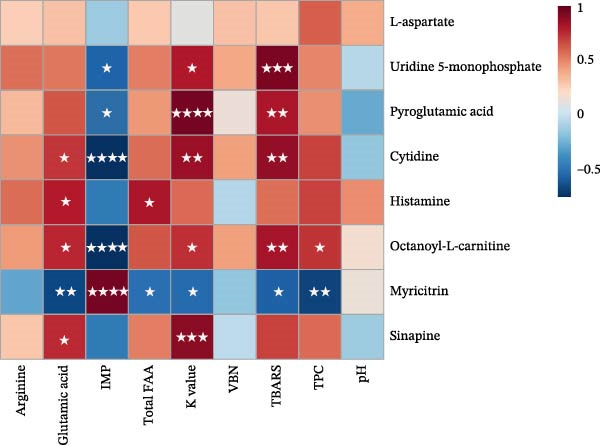
(b)

In wet‐aged fish fillets, UMP, cytidine, and pyroglutamic acid showed strong positive correlations with TBARS and K‐value (*p* < 0.05 to *p* < 0.0001), indicating they may be involved in lipid oxidation and nucleotide degradation. Conversely, myricitrin exhibited significant negative correlations with TPC, TBARS, glutamic acid, and K‐value (*p* < 0.05 to *p* < 0.0001), suggesting it could have antioxidant or preservative effects. These findings imply that specific metabolite profiles are closely associated with the quality changes during wet aging (Figure 4b).

## 4. Discussion

This study demonstrated that low‐temperature aging modifies the physicochemical properties of grouper fillets, primarily driven by endogenous proteolysis and moisture dynamics. During aging, the degradation of myofibrillar structures reduced the cellular WHC, leading to significant moisture loss. The crude fat content initially showed a relative increase due to the concentration effect of moisture loss but subsequently decreased as lipid oxidation and degradation progressed over aging time. In addition, the changes in fish muscle proteins during dry aging effectively improved moisture retention. Consequently, the formation of a dehydrated surface layer in dry‐aged grouper fillets minimized moisture loss during aging, resulting in a lower cooking loss than in wet‐aged fillets.

In addition to water retention, the aging process also significantly affected the texture of the fish fillets. During dry aging, the fillets’ hardness exhibited a two‐stage change. It first decreased through day 4 and then increased afterward. The softening in the early stage was mainly caused by endogenous proteolysis. However, the increased hardness from day 4 onward was likely due to severe moisture loss and protein aggregation. On the other hand, the hardness of wet‐aged fillets increased continuously over time. This different trend in wet aging might be attributed to oxidative changes and protein cross‐linking during storage [24]. These changes might be enhanced by the higher moisture content in the muscle fibers.

These texture changes are supported by the degradation of myofibrillar proteins. Continuous proteolysis, especially the breakdown of MHC, plays an important role in destroying the myofibrillar structure and softening the muscle. In our SDS‐PAGE results, the intensity of the MHC band decreased significantly during dry aging. This indicates a breakdown of the protein network, which may have contributed to the initial tenderization. By contrast, the wet‐aged fillets showed less MHC degradation and increased tropomyosin. Because higher levels of tropomyosin can reduce gel strength and elasticity [25], its accumulation during wet aging might explain why the texture changes differed from those in the dry‐aged group.

Although protein degradation was observed, the tenderization process in aged fish is fundamentally different from that in red meats. Unlike beef and other red meats, fish muscle is characterized by higher protease activity [1], a more neutral pH, and is susceptible to lipid oxidation [4]. Given the inherently softer initial texture of fish, the aging process therefore does not result in the significant structural breakdown and tenderization typically required for red meats. Instead, the final texture of aged fish, especially during dry aging, results from a combination of enzymatic softening and dehydration‐induced hardening.

Flavor development in aged fish is related to the accumulation of FAAs and nucleotide degradation products. Glutamic acid is a key amino acid responsible for the umami taste [26]. Its increase during aging is mainly caused by the proteolytic degradation of muscle proteins, driven by endogenous enzymes and microbial enzymes [26, 27]. In addition to FAAs, adenosine 5’‐monophosphate and IMP are well recognized as crucial compounds for umami flavor [28]. Importantly, IMP can act synergistically with umami‐enhancing amino acids like glutamic acid and aspartic acid. Because 5’‐nucleotides and amino acids bind to the same umami receptors, this synergistic effect significantly amplifies flavor perception [23]. However, further breakdown of IMP leads to the formation of hypoxanthine, which contributes to a bitter taste [29].

In this study, marked variations in the accumulation of FAAs and nucleotides were observed between the dry‐ and wet‐aged samples. The results demonstrate that dry aging more effectively preserves these umami‐related components than wet‐aged fish, because wet‐aging conditions facilitated a faster breakdown of nucleotides such as IMP, resulting in a lower concentration over aging time [30]. Therefore, by facilitating the accumulation of FAAs and IMP, dry aging provides a more effective approach to enhancing the flavor profile of fish fillets in this study. Although umami and sweet amino acids were the predominant taste‐active components, an accumulation of bitter compounds, such as hypoxanthine, proline, and lysine, was also observed. Even though their TAVs were relatively low compared to glutamate, these compounds might still influence the overall taste balance of the aged grouper fillets.

According to the Spearman correlation analysis, key metabolites in the dry‐aged fillets (such as mannitol and pyridoxine) exhibited strong positive correlations with glutamic acid, IMP, and total FAAs. This indicates a close association between these metabolites and the development of umami. Among the identified metabolites, mannitol was specifically elevated in dry‐aged grouper fillets in this study. As a sugar alcohol produced by enzymes, mannitol provides a slight sweetness and exhibits antioxidant properties, which potentially help proteins from oxidative degradation [31]. Similarly, pyridoxine (vitamin B6) increased during dry aging. Because certain microorganisms synthesize pyridoxine using the nitrogen from glutamine or glutamate [32], this increase may be attributed to microbial synthesis. Pyridoxine is involved in amino acid metabolism and is known to be an antioxidant; its accumulation might contribute to the biochemical stability of dry‐aged grouper fillets. Additionally, positive correlations were observed between histamine, inosine, and quality indicators (IMP, TBARS, and TPC), suggesting that nucleotide breakdown and microbial growth occur concurrently with certain textural and physicochemical changes.

By contrast, a different correlation pattern was observed in the wet‐aged fillets. Metabolites such as L‐aspartate, UMP, cytidine, and pyroglutamic acid were positively correlated with the FAA content, TBARS, and K‐value. This points to a simultaneous process of flavor compound accumulation and nucleotide degradation. The accumulation of amino acids such as aspartic acid during postmortem aging is known to enhance the brothy and umami flavor characteristics of the meat [2]. Furthermore, the rise in carnitine is likely associated with fatty acid metabolism and flavor development under wet‐aging conditions [33]. Another flavor‐related compound, pyroglutamic acid, was also detected in the samples. This naturally derived amino acid derivative is enzymatically converted into glutamate by 5‐oxoprolinase. Although it is less frequently discussed in meat aging studies, pyroglutamic acid is recognized for contributing to salty, umami, and sour flavor characteristics.

Regarding meat quality and safety indicators, histamine levels gradually increased during aging. Histamine concentration is commonly used as an indicator of fish quality, particularly in species with a high histidine content [34]. Typically, histamine is formed through the microbial decarboxylation of histidine [35]. Therefore, a proportional reduction in histidine, accompanied by a corresponding rise in histamine, might be expected. However, the data from this study did not show a strict inverse correlation between them. These results suggested that the dynamic changes in these amino acids during aging involve multiple complex metabolic pathways.

Overall, these correlation results indicated that the two aging methods involve distinct metabolic pathways, leading to different flavor profiles and oxidative risks. Our findings suggest that dry aging primarily relies on a concentration‐ and hydrolysis‐based mechanism. Moisture loss concentrates flavor precursors, such as FAAs and IMP, while specific microbial metabolites further increase the flavor’s complexity. In contrast, although wet aging retains more moisture and preserves product yield, it also accelerates nucleotide degradation, resulting in a different pathway for flavor development.

Finally, several limitations of the present study should be addressed. Although total bacteria counts were monitored, psychrotrophic bacteria are the primary microorganisms responsible for spoilage at low temperatures. Therefore, future studies should assess the food safety of aged fish products. In the PCA results, the cumulative variance explained by PC1 and PC2 was 32.5%, mainly due to the small sample size. While the statistical significance of the metabolomic data was confirmed through multiple analyses, the predictive capability remains limited by the sample size. Thus, larger sample cohorts are necessary in future research to better validate potential biomarkers. Additionally, although TAV indicated flavor changes, human sensory evaluation and consumer acceptance still need to be assessed in future investigations. In this study, the aging period was limited to 8 days because of rapid weight loss and elevated VBN levels. Future research should explore whether optimized environmental conditions could allow for longer aging periods to enhance flavor profiles without excessive yield loss. Moreover, quantifying specific volatile oxidation products would provide deeper insights into lipid oxidation pathways and further elucidate the oxidative stability of aged fish fillets. Overall, optimizing aging parameters and monitoring key metabolites are essential to balance flavor development with product yield and stability. These findings offer a practical scientific basis for the seafood industry to improve the overall quality and commercial viability of aged fish products.

## 5. Conclusion

This study showed that both dry and wet aging improve the physicochemical and flavor qualities of grouper fillets. Additionally, dry aging for 4 days was suggested as a practical balance between flavor development and product yield. At day 4 of dry aging, key flavor compounds such as IMP and FAAs increased in the grouper fillets. Extending the aging period further caused excessive weight loss without additional flavor benefits, making it less practical for industrial use. Moreover, dry aging led to the accumulation of mannitol and pyridoxine, which further enhanced flavor development. Conversely, wet aging increased levels of L‐aspartate and pyroglutamic acid, contributing to brothy‐umami flavors but also causing oxidative changes. By day 8, indicators such as TBARS and TPC remained within acceptable ranges, suggesting adequate oxidative and microbial stability. Overall, this study demonstrates that both aging methods can enhance flavor development through different pathways; however, careful monitoring is necessary to maintain quality and safety in aged grouper fillet production.

## Author Contributions

**Qin-Shan Yu**: conceptualization, investigation, methodology, data curation, validation, writing – original, writing – review and editing draft. **Hsiu-Ming Liu and Huey-Jine Chai**: conceptualization, methodology, funding acquisition. **Hsin-Hui Su**: writing – original, writing – review and editing draft. **Yung-Tsung Chen**: conceptualization, investigation, methodology, data curation, validation, writing – original, writing – review and editing draft, funding acquisition.

## Funding

This study was supported by the Fisheries Research Institute, Ministry of Agriculture, R.O.C. (Project ID: 112 AS‐6.3.2‐AI‐A2).

## Conflicts of Interest

The authors declare no conflicts of interest.

## Data Availability

The data that support the findings of this study are available from the corresponding author upon reasonable request.
